# A Highly Elastic and Fatigue‐Resistant Natural Protein‐Reinforced Hydrogel Electrolyte for Reversible‐Compressible Quasi‐Solid‐State Supercapacitors

**DOI:** 10.1002/advs.202000587

**Published:** 2020-06-05

**Authors:** Jingya Nan, Gaitong Zhang, Tianyu Zhu, Zhongkai Wang, Lijun Wang, Hongsheng Wang, Fuxiang Chu, Chunpeng Wang, Chuanbing Tang

**Affiliations:** ^1^ Institute of Chemical Industry of Forest Products Chinese Academy of Forestry Key Laboratory of Biomass Energy and Material, Jiangsu Province Nanjing Jiangsu 210042 China; ^2^ Department of Chemistry and Biochemistry University of South Carolina Columbia SC 29208 USA; ^3^ Co‐Innovation Center of Efficient Processing and Utilization of Forest Resources Nanjing Forestry University Nanjing Jiangsu 210037 China; ^4^ Biomass Molecular Engineering Center Anhui Agricultural University Hefei Anhui 230036 China

**Keywords:** elastic, hydrogel electrolytes, proteins, reversible compressibility, supercapacitors

## Abstract

Compressible solid‐state supercapacitors are emerging as promising power sources for next‐generation flexible electronics with enhanced safety and mechanical integrity. Highly elastic and compressible solid electrolytes are in great demand to achieve reversible compressibility and excellent capacitive stability of these supercapacitor devices. Here, a lithium ion‐conducting hydrogel electrolyte by integrating natural protein nanoparticles into polyacrylamide network is reported. Due to the synergistic effect of natural protein nanoparticles and polyacrylamide chains, the obtained hydrogel shows remarkable elasticity, high compressibility, and fatigue resistance properties. More significantly, the supercapacitor device based on this hydrogel electrolyte exhibits reversible compressibility under multiple cyclic compressions, working well under 80% strain for 1000 compression cycles without sacrificing its capacitive performance. This work offers a promising approach for compressible supercapacitors.

## Graphical abstract

A highly elastic hydrogel electrolyte with an ionic honeycomb‐like cellular structure is reported by integrating soy protein isolate nanoparticles into a polyacrylamide network. The hydrogel shows high elasticity, resilience, and fatigue resistance simultaneously. The hydrogel electrolyte‐based supercapacitor demonstrates reversible compressibility and electrochemical stability under multiple cyclic compressions. This work paves a way for advanced energy storage devices.

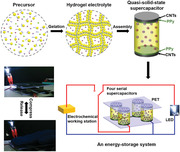

## 1 Introduction

Flexible electronic devices are attracting tremendous attention due to their excellent flexible, lightweight, and anti‐deformation properties.^[^ ^1^, ^2^, ^3^ ^]^ Corresponding energy‐storage devices are demanded to accommodate various mechanical deformations while maintaining electronic performances.^[^ ^2^, ^4^, ^5^ ^]^ Currently, flexible supercapacitors stand out as promising power units owing to their deformability, high power density, fast charge/discharge rates, and long cycle life, compared with conventional capacitors and batteries.^[^ ^6^, ^7^, ^8^, ^9^, ^10^, ^11^, ^12^ ^]^ In general, an ideal flexible supercapacitor should be bendable, foldable, stretchable, or compressible without sacrificing its high‐quality capacitive performance over long deformation cycles.^[^ ^1^ ^]^ Particularly, compressible supercapacitors, which should be elastic, resilient, and durable under repetitive compression deformations, are increasingly needed,^[^ ^13^, ^14^, ^15^ ^]^ considering that crashing and extruding damages for electronics frequently occur in practical applications. In recent years, much progress has been made to develop compressible supercapacitors, focusing primarily on the fabrication of compressible electrodes, including compressible substrates coated with a thin layer of electrochemically active materials,^[^ ^16^ ^]^ and free‐standing conductive carbon‐based materials with a sponge‐like structure (mainly including graphene and carbon nanotube based aerogels^[^ ^17^, ^18^ ^]^ and foams^[^ ^15^, ^19^ ^]^). Despite recent advances, there are two major limitations. First, because liquid electrolytes are usually used in these supercapacitors, these devices are liable to suffer from the leakage of harmful electrolytes and undesired dislocation of electrodes when being repeatedly compressed. Second, fabrication of devices involves incompressible packaging materials, hence hindering the mechanical integrity.

To overcome these limitations, supercapacitors based on solid electrolytes are favored, owing to their facile packaging, simple configuration, mechanical integration, and enhanced safety without potential leakage of toxic liquid electrolytes under harsh mechanical conditions.^[^ ^1^ ^]^ At present, the most widely used polyvinyl alcohol‐based gel electrolytes for solid‐state supercapacitors are neither very elastic nor compressible.^[^ ^1^, ^12^ ^]^ Consequently, the compressibility and capacitive performance of these devices are largely limited by the electrode materials.^[^ ^13^, ^15^, ^19^ ^]^ For example, the nonconductive substrates would affect the charge‐discharge rates;^[^ ^13^ ^]^ the intrinsic hydrophobicity and high thickness of sponge electrode materials result in high internal resistance. These drawbacks severely limit the electrochemical performance of the devices. Several recent work has successfully developed elastic hydrogel electrolytes, including Agar/hydrophobically associated polyacrylamide hydrogel electrolyte^[^ ^20^ ^]^ and Al‐alginate/polyacrylamide hydrogel electrolyte,^[^ ^21^ ^]^ to achieve supercapacitors with superior elasticity and toughness at the device level. Thus, it is highly sought to explore novel solid electrolytes with excellent conductivity, high elasticity and fatigue resistance that can be utilized in solid‐state supercapacitors with reversible compressibility and capacitive stability.

Hydrogels can serve as ionic conductors that have been widely used in sensors,^[^ ^22^, ^23^ ^]^ actuators,^[^ ^24^ ^]^ and touch panels.^[^ ^25^ ^]^ Thus, hydrogels possessing ion conduction, semi‐solid state, and excellent mechanical performance are promising candidates as quasi‐solid electrolytes for compressible supercapacitors.^[^ ^26^ ^]^ However, most hydrogels are considered as mechanically weak materials,^[^ ^27^ ^]^ thus largely limiting their applicability. In the last 15 years, various strategies have been exploited to strengthen and toughen hydrogels. Double‐network hydrogels are emerging materials, in which the brittle networks act as sacrificial bonds to rupture and dissipate energy, making the whole materials tough.^[^ ^28^, ^29^ ^]^ Nanocomposites utilize inorganic nanoparticles as cross‐linkers and stress buffers to dissipate energy.^[^ ^30^, ^31^ ^]^ Polyampholyte materials rely on weak ionic bonds to reversibly break and re‐form, dissipating energy, and enhance the fatigue resistance.^[^ ^32^ ^]^ Hybrid hydrogels that mix covalent bonds and non‐covalent bonds allow the fractured bonds to be re‐formed, realizing toughness with partial or full self‐recovery after internal damage.^[^ ^33^, ^34^ ^]^ Therefore, introduction of efficient energy‐dissipating mechanisms into cross‐linked networks is crucial for toughing hydrogels, which effectively prevent crack propagation upon loading.^[^ ^28^, ^29^, ^34^, ^35^, ^36^ ^]^

Herein, we report a highly elastic hydrogel electrolyte with an ionic honeycomb‐like cellular structure, in which polyacrylamide (PAAm) chains are cross‐linked to form a cellular structure, and soybean protein isolate (SPI) nanoparticles are coagulated around the polymer chains to form cell walls. The synergistic effect of SPI nanoparticles and PAAm chains endows the hydrogel with high elasticity, resilience, and fatigue resistance simultaneously. A symmetric supercapacitor is then fabricated by using the elastic hydrogel as a quasi‐solid‐state electrolyte and two polypyrrole‐coated carbon nanotubes papers (PPy‐coated CNTs) as electrodes. The resultant device demonstrates excellent electrochemical performance, delivering a specific capacitance of 246.8 F g^−1^ at 0.3 A g^−1^ with a high rate capability of 62.5% (from 0.3 to 12 A g^−1^), a maximum energy density of 21.4 Wh kg^−1^ and a maximal power density of 2580 W kg^−1^, as well as remarkable cycling stability (≈80% capacitance retention after 5000 charge/discharge cycles). In addition, the device can be intrinsically compressed to 80% strain with pressure‐induced capacitance enhancement, largely owing to the highly elastic and fatigue‐resistant hydrogel electrolyte. More importantly, the device can undergo multiple cyclic compressions and maintain high capacitance retention for 1000 compression cycles even at 80% strain, without structural damage and electrochemical failure.

## 2 Results and Discussion

### 2.1 Fabrication of SPI‐PAAm Hydrogel

**Figure 1:**
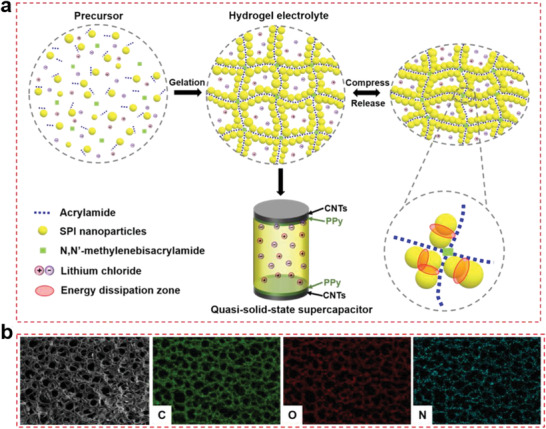
Fabrication of SPI‐PAAm hydrogel. a) Schematic of preparation of the SPI‐PAAm hydrogel with ammonium persulfate (APS, initiator), *N,N*′‐methylenebisacrylamide (MBAA, crosslinker) and lithium chloride (LiCl, charge carrier), as well as the construction of hydrogel electrolyte‐based supercapacitors with two PPy‐coated CNTs electrodes directly paving on each side of the electrolyte. b) Energy dispersive X‐ray spectroscopic mapping images show the elemental distribution of C, O, and N on the cross section of freeze‐dried hydrogel.

The hydrogel was fabricated by integrating two types of macromolecules. Coagulation of SPI nanoparticles was followed by radical polymerization of acrylamide, as shown in **Figure** **1**a. We used a heating method^[^ ^37^ ^]^ to induce SPI powders dispersed in deionized water (Figure S1a, Supporting Information). SPI nanoparticles with an average diameter of ≈70 nm were obtained, showing a globular structure (Figure S1b,c, Supporting Information). In the dispersion solution, SPI nanoparticles comprising amino groups (N‐terminal) and carboxyl groups (C‐terminal) were negatively charged under neutral pH (Figure S1d, Supporting Information),^[^ ^38^, ^39^ ^]^ which induced repulsive force between the nanoparticles for stabilization. Lithium chloride (LiCl) salts were then dissolved in the dispersion serving as an ionic charge carrier and a volatilization inhibitor, which would allow hydrogel to be conductive and dry slowly.^[^ ^25^ ^]^ A mixed solution of acrylamide and SPI dispersion was used as the precursor (unless otherwise stated, the water content was fixed at 80 wt%). Acrylamide monomers (AAm) were first polymerized to form a 3D network in the presence of ammonium persulfate (APS) as the initiator, *N,N*′‐methylenebisacrylamide (MBAA) as the crosslinker, and *N*,*N*,*N*′,*N*′‐tetramethylethylenediamine (TEMED) as the accelerator. The negatively charged SPI nanoparticles were aggregated with the positively charged polyacrylamide (PAAm) chains through electrostatic attraction, and coagulated by van der Waals interactions.^[^ ^40^, ^41^, ^42^ ^]^ Thus, the lithium ion‐containing SPI‐PAAm hydrogel had a honeycomb‐like cellular structure, in which PAAm chains were cross‐linked to form a cellular structure with the cell dimensions in the order of tens of micrometers and SPI nanoparticles were coagulated around the polymer chains to form cell walls, as indicated by Scanning Electron Microscopy (SEM) (Figure 3a–c). C, O, and N elements were homogeneously distributed on the network skeleton, revealing the uniform coagulation of SPI nanoparticles around PAAm chains (Figure 1b). Furthermore, the fluorescence images (Figure S2, Supporting Information) demonstrated that the fluorescent coagulations of SPI (shown in red) were uniformly distributed within the hydrogel. It is worth noting that PAAm chains were formed by radical polymerization before coagulation of SPI nanoparticles.

### 2.2 Mechanical Properties of Hydrogel

**Figure 2:**
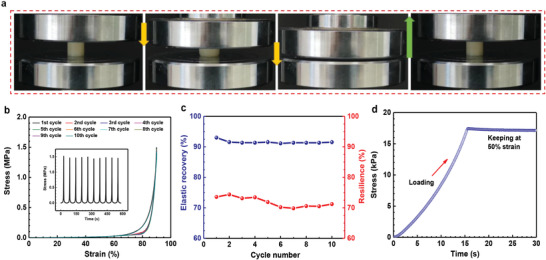
Mechanical compressibility and elasticity of the hydrogel. a) Photographs of the hydrogel showing high compressibility and elasticity under 90% strain. b) Stress–strain curves of the hydrogel under successive compression cycles and the inset is the corresponding stress–time curves. c) Changes of elastic recovery and resilience as a function of compression cycles for the hydrogel. d) Stress–time curve in a static compression test shows that when the hydrogel was compressed at 50% strain and held for a few seconds, the induced stress could be maintained over time without relaxation.

As expected, the as‐fabricated SPI‐PAAm hydrogel (AAm:SPI = 1:0.096) exhibited high compressibility and elasticity. It rapidly recovered to its original shape upon 90% compressive strain without structural collapse or damage during ten successive uniaxial compression cycles (**Figure** **2**a and Movie S1, Supporting Information). The cyclic stress–strain curves of the hydrogel are shown in Figure 2b. Notably, the crescent‐shaped stress–strain curve shows two distinct deformation regions: an initial plateau region in which the stress nonlinearly increased to ∼30 kPa at 60% strain, and a subsequent densification region where the stress increased sharply to ∼1.5 MPa at 90% strain. Upon unloading, the stress dropped sharper than during compression with a small hysteresis. The hysteresis loop in each stress–strain curve was found to be very narrow, indicating that there was little energy dissipated during compress–release cycles. This is noticeably different from traditional viscoelastic materials, which generally dissipate much more energy in viscoelasticity‐induced hysteresis. The changes of elastic recovery and resilience as a function of compression cycles are shown in Figure 2c. During 10 compression cycles at 90% strain, the elastic recovery and resilience values of the hydrogel retained at more than 90% and 70%, respectively. The high elasticity was resulted from the synergistic effect of SPI nanoparticles and PAAm chains. The introduction of SPI nanoparticles reinforced the stiffness of network, thus increasing the elastic recoil ability of polymer chains. Besides, water serves as a plasticizer to reduce the inter/intra‐chain friction, allowing them to recoil without considerable energy dissipation. Moreover, the remarkable elasticity was further corroborated by a static compression test (Figure 2d), in which the induced stress could be remained over time when the hydrogel was compressed and held at 50% strain, differing far from the representative stress‐relaxation behavior usually observed in viscoelastic materials,^[^ ^43^, ^44^, ^45^, ^46^, ^47^ ^]^ but resembling the typical elastic behavior of rubber.^[^ ^48^, ^49^ ^]^

**Figure 3:**
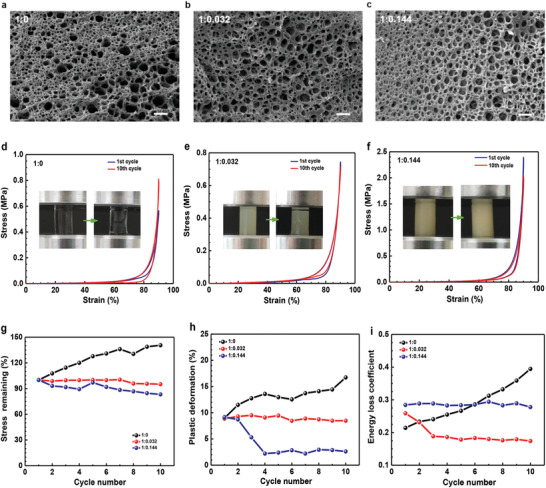
Morphology and mechanical behavior. a–c) SEM cross section images of three hydrogels with different mass ratios of AAm to SPI. The scale bars: 50 µm. d–f) Stress–strain curves of hydrogels under cyclic compression, the insert photos show the corresponding samples. g–i) Changes of maximum stress, plastic deformation, and energy loss coefficient for hydrogels during the successive compression cycles at 90% strain.

To further investigate the synergistic effect of SPI nanoparticles and PAAm chains, we prepared a series of SPI‐PAAm hydrogels by adjusting the mass ratios of AAm to SPI. **Figure** **3**a–c shows that SPI contents mainly determined the thickness of cellular walls, due to the coagulation of SPI nanoparticles around the PAAm chains. Cyclic compression tests were shown in Figure S4, Supporting Information. Pure PAAm hydrogel with a thin‐wall structure was severely damaged upon cyclic compression and displayed incremental plastic deformation and energy dissipation (Figure 3d,g–i), indicating that the single PAAm polymer chains were susceptible to rupture because there was no mechanism to disperse the applied stress.^[^ ^28^, ^34^, ^35^, ^50^, ^51^ ^]^ Meanwhile, the stress enhancement was ascribed to the densification of the network structure. With the increase of SPI content, the hybrid hydrogel with a thick‐wall structure suffered from slight crack, exhibiting small stress reduction, low plastic deformation and little energy dissipation (Figure 3e,g–i). When further increasing SPI content, the hybrid hydrogel with a thicker‐wall structure could tolerate large deformation and displayed lower plastic deformation and relatively more energy dissipation (Figure 3f,g–i). The increased elastic recovery (equal to the decreased plastic deformation) with the increase of SPI content was mainly attributed to the reinforced elastic recoil of PAAm chains; the more energy dissipation with the increase of SPI content probably oriented from the sliding friction between SPI nanoparticles and plastic deformation of SPI nanoparticles. In addition, compression tests of hybrid hydrogels with different mass ratios of AAm to SPI (Figure S5, Supporting Information) show that the compressive strength of hydrogels at 90% strain first increased dramatically and then decreased while increasing the SPI content, indicating that there should be a threshold for SPI content to enhance the compressive strength of the hydrogel. The rheological tests (Figure S6, Supporting Information), in which the storage modulus and loss modulus of these hydrogels presented a first increasing and then decreasing trend with the increase of SPI contents, further revealed that SPI nanoparticles had a synergistic effect on the mechanical properties of the hydrogels. On one hand, SPI nanoparticles could serve as a robust filler to reinforce the network skeleton; On the other hand, the sliding friction between SPI nanoparticles and plastic deformation of SPI nanoparticles could effectively disperse the applied stress and dissipate energy, endowing the hydrogel with remarkable elasticity and toughness, as illustrated in Figure 1a.

### 2.3 Fatigue Resistance

**Figure 4:**
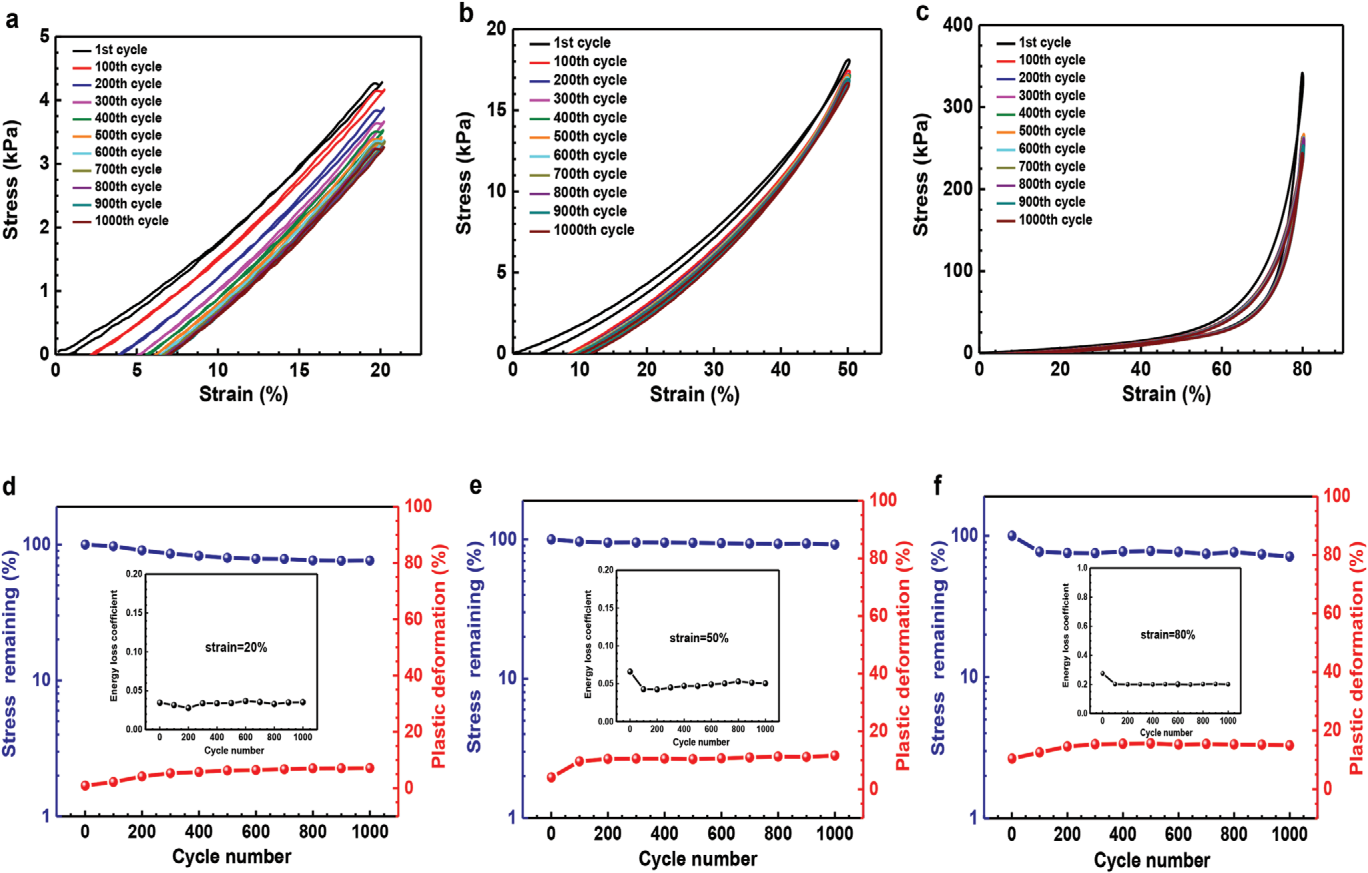
Fatigue resistance. a–c) Stress–strain curves of hydrogel with a mass ratio of AAm to SPI at 1:0.144, compressed for 1000 cycles at 20% strain, 50% strain, and 80% strain, respectively. d–f) Changes of maximum stress, plastic deformation and energy loss coefficient during 1000 cycles at 20% strain, 50% strain, and 80% strain, respectively.

We further investigated the fatigue resistance of the as‐prepared hydrogel (AAm:SPI = 1:0.144), which was compressed at different strains for multiple successive cycles. During cyclic tests, the water content of the hydrogel remained at ∼80 wt% with a silicone oil‐seal treatment. The hydrogel possessed outstanding fatigue resistance as shown in **Figure** **4**. After 1000 compression cycles at 20% strain, the hydrogel retained over 76% of maximum stress and only experienced ≈7% height reduction (Figure 4a,d). While at 50% strain for 1000 cycles, the hydrogel showed a reduction of maximal stress at ≈8% and suffered ≈11% plastic deformation (Figure 4b,e). Even with a large deformation of 80% strain for 1000 cycles, the hydrogel still exhibited excellent cyclic compressible performance, maintaining over 71% of its maximum stress and showing height retention of ≈84% (Figure 4c,f). It should be noted that the aforementioned parameters mainly changed within the initial 400 cycles and then gradually stabilized in the subsequent 600 cycles, indicating that the hydrogel could withstand a large number of cycles without further fatigue damage. Moreover, the small energy loss coefficient (below 0.3) (Figure 4d–f) also reflected that the hydrogel was negligibly damaged under multiple cyclic compressions.

### 2.4 Electrochemical Performance of Hydrogel Electrolyte‐Based Supercapacitors

The as‐formed SPI‐PAAm hydrogel was expected to serve as an electrolyte for quasi‐solid‐state supercapacitors, due to the presence of dissolved LiCl salts.^[^ ^22^, ^23^, ^25^ ^]^ The hydrogel electrolytes possessed high ionic conductivity in the range of 2.44–16.34 mS cm^−1^ (Figure S7a, Supporting Information), higher than most gel electrolytes (0.1–1 mS cm^−1^)_._^[^ ^1^ ^]^ Notably, the ionic conductivity of hydrogel electrolytes with different SPI contents revealed that the ionic conductivity increased within a certain range of SPI contents, while higher SPI content afforded a decrease in ionic conductivity, suggesting that the structure of polymer matrix had a significant effect on ion mobility.^[^ ^52^, ^53^ ^]^ It should be mentioned that without LiCl salts both PAAm hydrogel and SPI‐PAAm hydrogel contribute negligible ionic conductivity (Figure S7b, Supporting Information). In addition, the ionic conductivity and equivalent series resistance of hydrogels with SPI and without SPI (Figure S7b,c, Supporting Information) suggested that the SPI colloids had little effect on the ionic and electrochemical properties of hydrogel electrolytes.

**Figure 5:**
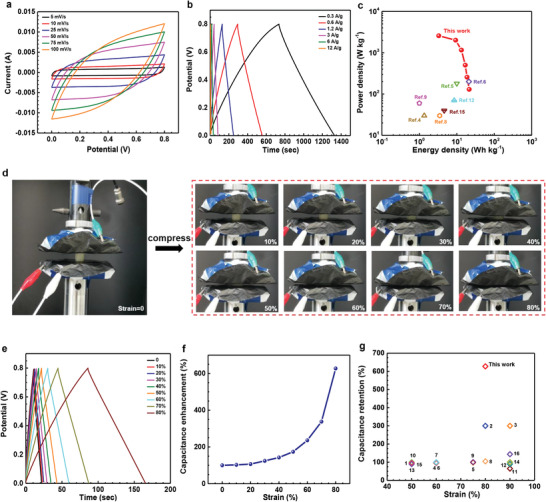
Electrochemical performance of a hydrogel electrolyte‐based supercapacitor. a) Cyclic voltammetry (CV) curves of the assembled supercapacitor at different scan rates from 5 to 100 mV s^−1^. b) Galvanostatic charge/discharge (GCD) curves of the assembled supercapacitor at various current densities from 0.3 to 12 A g^−1^. c) Ragone plot of the assembled supercapacitor device. References in this figure corresponds to the relevant references shown in Table S1, Supporting Information. d) Photographs of the compressible supercapacitor at different strain levels from 0% to 80%. e) GCD curves under different strains from 0% to 80% at a charging/discharging current density of 6 A g^−1^. f) Capacitance enhancement (%) calculated from GCDs of (e). g) Capacitance retention of the compressible supercapacitor and other previously reported devices. Numbers in this figure corresponds to the relevant references shown in Table S1, Supporting Information.

We selected a hydrogel with AAm:SPI = 1:0.144 as the electrolyte to fabricate a quasi‐solid‐state supercapacitor with PPy‐coated CNTs papers as electrodes. Due to the likely hydrogen bonding between hydrogel electrolyte and PPy, the hydrogel electrolyte easily adhered to the two identical electrodes in the symmetric supercapacitor without the use of extra binders, separators and current collectors (Figure 1a and Figure S8, Supporting Information). For the electrodes, PPy was the main electrode material and uniformly coated on the CNTs paper by electrochemical deposition, while the thin CNTs paper was used as both a current collector and scaffold for supporting the active material (Figure S9, Supporting Information). Electrochemical performance of the assembled symmetric supercapacitor was analyzed in a voltage window of 0.8 V. **Figure** **5**a,b shows cyclic voltammetry (CV) curves at different scan rates and galvanostatic charge/discharge (GCD) curves at various current densities. CVs maintained near‐rectangular shape at scan rates from 5 to 100 mV s^−1^, demonstrating that the assembled device could endure fast voltage/current change rates. GCDs exhibited an isosceles triangular shape at current densities from 0.3 to 12 A g^−1^, and there was no obvious voltage drop (IR drop) observed, indicating that the device had low internal resistance. This attributed to the excellent interfacial contact of the hydrogel electrolyte and the thin thickness of PPy‐coated CNTs electrodes.^[^ ^54^ ^]^ The corresponding specific capacitances calculated from both CVs and GCDs were plotted in Figure S10, Supporting Information. The device achieved a specific capacitance of 246.8 F g^−1^ at 0.3 A g^−1^ and remained 154.3 F g^−1^ at 12 A g^−1^, showing a high rate capability of 62.5%. Besides, the capacitances decreased with the increase of scan rates and current densities, which originated from the ion diffusion limitation. The Ragone plot (Figure 5c) shows that the symmetric supercapacitor delivered a maximum energy density of 21.4 Wh kg^−1^ with a power density of 130 W kg^−1^, and maintained at 3.3 Wh kg^−1^ with a high power density of 2580 W kg^−1^. These values are much higher than previously reported compressible supercapacitor devices (Figure 5c), placing within the region of standard supercapacitors.^[^ ^55^ ^]^ In contrast, a supercapacitor with bare CNTs as electrodes possessed a low capacitance of 34 F g^−1^ at 0.3 A g^−1^ (Figure S11, Supporting Information), indicating that PPy coating significantly improved the capacitive performance. At the same time, the single PPy‐coated CNTs electrode achieved a remarkable specific capacitance of 987.3 F g^−1^ at 0.3 A g^−1^ (Figure S10b, Supporting Information), which is comparable to or even higher than previously reported conducting polymer‐based composite electrodes with liquid electrolytes.^[^ ^54^, ^56^ ^]^ In addition, the supercapacitor device with PPy‐coated CNTs electrodes exhibited good cycling stability of ≈80% capacitance retention after 5000 charge/discharge cycles (Figure S12, Supporting Information), suggesting that the structural stability of PPy was enhanced by interweaved CNT nanowires, thus avoiding its mechanical degradation by swelling and shrinkage during doping and dedoping process. It was believed that the high ionic conductivity of the hydrogel electrolyte and the synergistic effect in hybrid electrodes contributed to the overall device performance.

### 2.5 Highly Compressible Supercapacitor

Owning to the high mechanical compressibility of the hydrogel electrolyte, the assembled supercapacitor was expected to be highly compressible. The device was compressed at different strain levels of 0–80% and its capacitive behavior was recorded, as shown in Figure 5d. Apparently, the device could endure large strains up to 80% without structural fracture or damage. The capacitive performance of the device was significantly enhanced as the strain increased from 0% to 80% (Figure 5e,f and Figure S13, Supporting Information). Both GCD and CV curves expanded with the increase of compressive strain (Figure 5e and Figure S13a, Supporting Information). The specific capacitance calculated from GCDs and CVs showed an improvement of ≈6.3‐fold (Figure 5f) and ≈6.4‐fold (Figure S13b, Supporting Information) at 80% strain, respectively. This capacitance retention is much higher than many previously reported devices (Figure 5g and Table S1, Supporting Information). This performance was attributed to the compression‐induced enhancement of ionic conductivity and acceleration of ion transfer at the electrode‐electrolyte interfaces.^[^ ^57^, ^58^ ^]^ On one hand, the storage modulus and loss modulus of the hydrogel electrolyte decreased with the increase of compression strains from 10% to 80% (Figure S14, Supporting Information), which resulted in the decrease of viscosity. According to the inverse relationship of viscosity and mobility of an electrolyte,^[^ ^57^, ^59^, ^60^ ^]^ the decrease of viscosity could increase the ion mobility and hence conductivity. This explanation was corroborated with compression‐induced enhancement of ionic conductivity with the increase of strain (Figure S15, Supporting Information). On the other hand, the increasing compression could improve the interfacial contact between the electrolyte and the electrodes, thus increase the accessible electrochemical sites and accelerate the ion transfer.^[^ ^31^, ^57^, ^58^ ^]^ Such increase could be observed by the decrease of the internal resistance of the device under compression (Figure S13c, Supporting Information). Therefore, the hydrogel electrolyte‐based supercapacitor could be highly compressible with compression‐induced capacitance enhancement. Although the capacitive behavior has negligible changes under various thicknesses of hydrogel electrolyte (Figure S16, Supporting Information), it is worth noting that thickness does have an influence on the energy density (specially based on volume), which could be studied in future work.

### 2.6 Capacitive Stability Under Cyclic Compression

**Figure 6:**
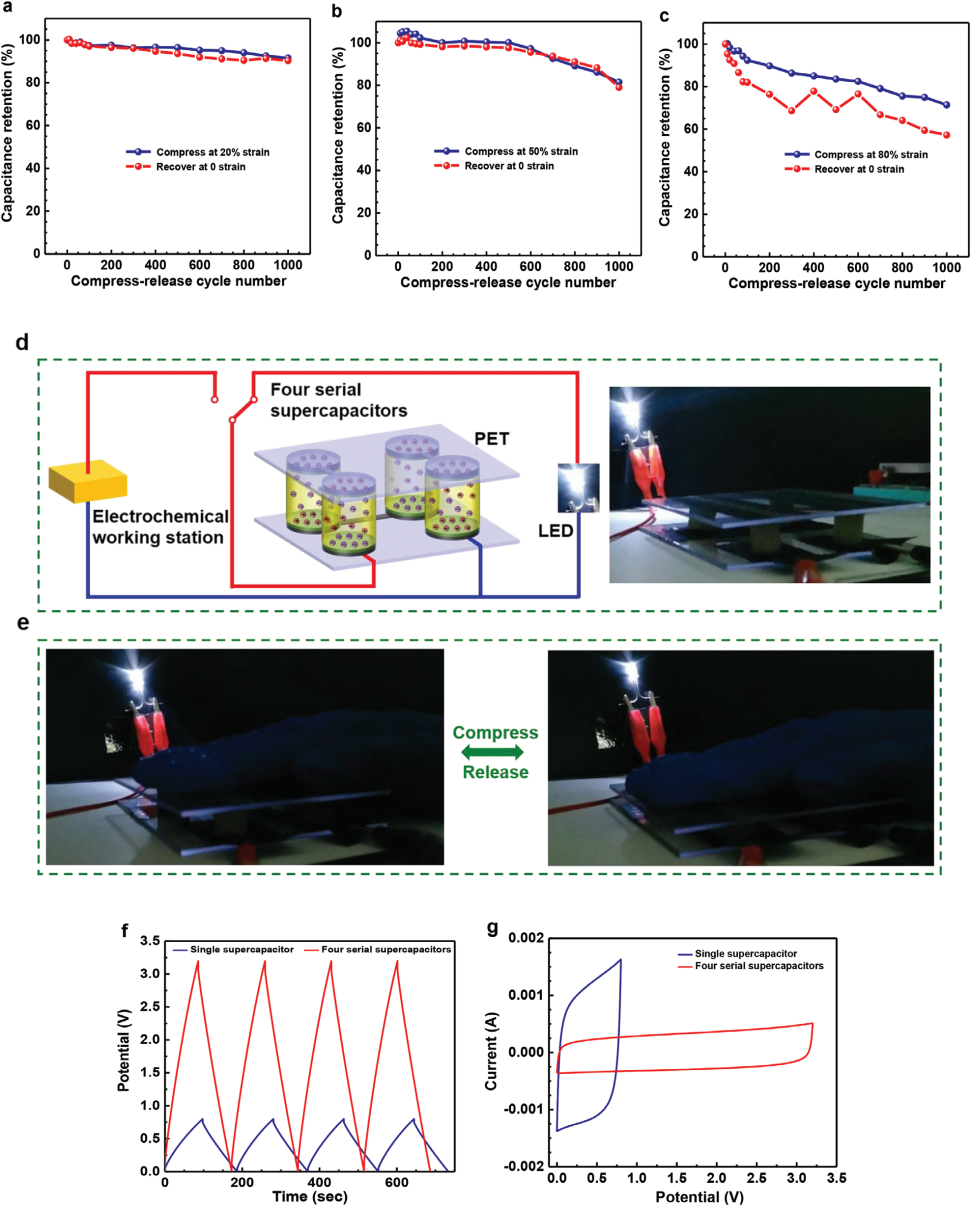
Capacitive performance under cyclic compression. a–c) Capacitance retention of the supercapacitor that was compressed for 1000 cycles at different strain levels of 20%, 50%, and 80%, respectively. d) Schematic of an energy‐storage system that four supercapacitors were connected in series as an integrated device to be charged through an electrochemical working station and then power a LED. e) Photographs of the integrated device that could be compressed reversibly and still powered well under cyclic compression. f) GCD curves of the single supercapacitor and four serial supercapacitors at current density of 1.2 A g^−1^. g) CV curves of the single supercapacitor and four serial supercapacitors at a scan rate of 10 mV s^−1^.

The outstanding elasticity and fatigue resistance of the hydrogel electrolyte could enable the supercapacitor to achieve reversible compressibility at a device level. As shown in Movies S2 and S3, Supporting Information, the device could be compressed and instantly recovered to its original shape during successive compress–release cycles, demonstrating excellent reversible compressibility. Meanwhile, the capacitive behaviors were investigated by compressing the device at different strain levels of 20%, 50%, and 80% for 1000 successive cycles, respectively. The device showed slight capacitance degradation (within ≈10%) at 20% strain for 1000 cycles (**Figure** **6**a); both CVs and GCDs nearly overlapped at each compressed and recovered state (Figure S17, Supporting Information). After being compressed at 50% strain for 1000 cycles, the device could maintain the capacitance retention of over 80% (Figure 6b and Figure S18, Supporting Information). Moreover, even under 80% strain for 1000 cycles, the device still remained a capacitance retention of ≈71% for the compressed state and ≈57% for the recovered state (Figure 6c and Figure S19, Supporting Information). The difference between the compressed state and the recovered state mainly resulted from the plastic deformation of the hydrogel electrolyte in the cyclic compression. In addition, as the device was exposed to the air, the prolonged dehydration of the hydrogel electrolyte could adversely impact the capacitance. Considering practical applications, four quasi‐solid‐state supercapacitors were connected in series to integrate an energy‐storage unit as depicted in Figure 6d. This integrated unit could effectively light up a white light‐emitting diode (LED, operating voltage: 3.0–3.4 V) when fully charged through an electrochemical working station (Figure 6d). More encouragingly, four serial devices could be compressed reversibly as one unit and still powered well under cyclic compression, as shown in Figure 6e and Movie S4, Supporting Information. Besides, both GCD and CV curves of the integrated device demonstrated an increased operating voltage (3.2 V) than that of a single device (0.8 V) (Figure 6f,g).

## 3 Conclusions

In summary, a hydrogel electrolyte with an ionic honeycomb‐like cellular structure was prepared by simply integrating SPI nanoparticles into a PAAm network. The introduction of SPI nanoparticles reinforced the network and effectively dissipated energy, thus endowing the hydrogel with high elasticity, resilience, and fatigue resistance simultaneously. By using the hydrogel as a quasi‐solid‐state electrolyte, the quasi‐solid‐state supercapacitor device was fabricated, demonstrating excellent electrochemical performance and high compressibility. More importantly, the device could undergo multiple cyclic compression and maintain high capacitance retention for 1000 compression cycles even at 80% strain, without structural damage and electrochemical failure. The demonstrated hydrogel electrolyte achieved remarkable reversible compressibility and capacitive stability at the device level, making it possible for the conventional electrode materials, such as porous carbon, conducting polymers, metal oxides, and their composites, etc. to meet the requirements of the compressible supercapacitors.

## 4 Experimental Section

##### Fabrication of SPI‐PAAm Hydrogel

The SPI‐PAAm hydrogel was fabricated by free radical polymerization of AAm and coagulation of SPI nanoparticles as shown in Figure 1a. Typically, SPI powders (1.2 g, Macklin) were first dispersed in deionized water (30 g) under vigorous stirring at 95 °C for 4 h, yielding a mixture of yellow dispersion. LiCl (1.9 g, Aladdin), AAm (6.3 g, Aladdin), APS (37.8 mg, Sigma‐Aldrich), and MBAA (18.9 mg, Sigma‐Aldrich) were then added into the dispersion and stirred for 1 h at room temperature. After degassing and removing dissolved oxygen, TEMED (15.75 mg, Sigma‐Aldrich) was added into the solution as the crosslinking accelerator. Next, the precursor was poured into a mold and cured in a water bath at 80 °C for 24 h. Finally, the SPI‐PAAm hydrogel was obtained by peeling out of the mold and removing the surface water.

##### Fabrication of the Quasi‐Solid‐State Supercapacitor

For making a quasi‐solid‐state supercapacitor, the as‐prepared hydrogel containing LiCl salts was used as the quasi‐solid electrolyte, and two identical PPy‐coated CNTs papers were served as electrodes. PPy were electrodeposited onto CNTs papers by a potentiostatic method. The electrochemical deposition was conducted in a three‐electrode configuration at 0 °C with a constant voltage of 0.8 V for 15 min, in which a solution of *p*‐toluenesulfonic acid (0.1 m, Aladdin), sodium toluenesulfate (0.3 m, Aladdin), and pyrrole monomer (v:v) (0.5%, Aladdin) worked as the electrolyte, and the CNTs paper (XFNANO, INC), platinum electrode, and saturated calomel electrode respectively served as the working electrode, counter electrode, and reference electrode. Before electrodeposition, pyrrole was distilled. Two identical PPy‐coated CNTs papers were directly paved on each side of the hydrogel electrolyte to form a sandwiched symmetric cell without extra binders, separators, or current collectors. Thus, a quasi‐solid‐state supercapacitor device was obtained.

##### Characterization

The structure of SPI nanoparticles was observed by a transmission electron microscopy (JEM 1400‐PLUS, JEOL) at an acceleration voltage of 80 kV. The hydrodynamic diameter and size distribution of SPI nanoparticles were tested using Malvern Nano‐ZS90. FTIR spectra were recorded on a Fourier transform infrared spectrometer (Nicolet iS10, Thermo Scientific) from 4000 to 400 cm^−1^ at room temperature. The morphology and structure of hydrogels were characterized via an SEM (EVO 18 Special Edition, ZEISS) equipped with an energy dispersive X‐ray spectroscopic detector. The ionic conductivity of hydrogel electrolytes was measured by a four‐probe method with FOUR PROBES TECH RTS‐9 at room temperature. Rheological tests of hydrogel electrolytes were performed using a rheometer (HAAKE RS6000, Thermo Scientific). Frequency sweeps from 0.01 to 10 Hz were carried out with different strain levels from 0% to 80% at room temperature.

##### Mechanical Testing

Compressive tests were performed using a universal testing machine (UTM 4304, Shenzhen SUNS) with a 1 kN load cell at room temperature. Cylindrical samples (13 mm in diameter and ≈15 mm in height) were mounted between the two flat‐surface compression stages without an initial loading. The samples were compressed and released along the uniaxial direction. The fatigue behaviors of the hydrogel were evaluated by conducting cyclic compression tests with 1000 successive loading‐unloading cycles between 20% and 80% strain. During the fatigue tests, the samples were sealed with silicone oil to minimize dehydration, so the water content of the hydrogel samples remained at ∼80 wt%. All the cyclic tests were conducted at a constant strain rate of 0.5 mm s^−1^. The elastic recovery, resilience, plastic deformation, and energy loss coefficient were calculated according to the method described in Figure S4d, Supporting Information.

##### Electrochemical Testing

The electrochemical measurements of the as‐assembled supercapacitor device were conducted on an electrochemical working station (CHI 760E, Shanghai Chenhua) at room temperature (Figure S8a, Supporting Information). CV and GCD curves were collected in a two‐electrode configuration. Electrochemical impedance spectra were measured at frequencies ranging from 0.01 to 5000 Hz with a potential amplitude of 5 mV. With the assistance of the universal testing machine, the device was compressed at different strains from 0% to 80%. At the same time, the electrochemical behavior of the device under different compressive strains was tested and recorded by the electrochemical working station. The capacitive stability of the device was evaluated by compressing the device at different strains (20%, 50%, and 80%) for 1000 successive cycles and by collecting the corresponding electrochemical behaviors simultaneously.

## Conflict of Interest

The authors declare no conflict of interest.

## Supporting information

Supporting Information

Supplemental Movie 1

Supplemental Movie 2

Supplemental Movie 3

Supplemental Movie 4
