# Supplementary material for: A Highly Elastic and Fatigue‐Resistant Natural Protein‐Reinforced Hydrogel Electrolyte for Reversible‐Compressible Quasi‐Solid‐State Supercapacitors
Source: Adv Sci (Weinh). 2020 Jun 5;7(14):2000587. doi: 10.1002/advs.202000587 (PMC7375230; doi:10.1002/advs.202000587)
Supplement: Supplementary file 1 — Supporting Information [file ADVS-7-2000587-s001.pdf]

Copyright WILEY-VCH Verlag GmbH & Co. KGaA, 69469 Weinheim, Germany, 2020.

Supporting Information

A Highly Elastic and Fatigue-Resistant Natural Protein-Reinforced Hydrogel Electrolyte for Reversible-Compressible Quasi-Solid-State Supercapacitors

Jingya Nan, Gaitong Zhang, Tianyu Zhu, Zhongkai Wang, Lijun Wang, Hongsheng Wang, Fuxiang Chu, Chunpeng Wang, and Chuanbing Tang**

1. Electrochemical Calculations

The specific capacitance (C_m, F g^{-1}) of the supercapacitor device is calculated from the GCD curves, according to Equation (S1):

$$C_m = \frac{I\Delta t}{m\Delta U} \qquad \text{(Equation S1)}$$

Where I is the discharge current, Δt is the discharge time, m is the total mass of active materials in two electrodes, ΔU is the voltage after IR drop.

The specific capacitance (C_m, F g^{-1}) of the device is also calculated from the CV curves, according to Equation (S2):

$$C_m = \frac{1}{mv\Delta U}\int_{U_-}^{U_+} i(U)\, dU \qquad \text{(Equation S2)}$$

Where $i(U)$ is the current, m is the total mass of active materials in two electrodes, v is the potential scan rate, ΔU is the voltage window.

The energy density (E, Wh kg^{-1}) and power density (P, W kg^{-1}) of the device are calculated according to Equations (S3,4):

$$E = \frac{1}{2} C_m \Delta U^2 \qquad \text{(Equation S3)}$$

$$P = \frac{E}{\Delta t} \qquad \text{(Equation S4)}$$

Where C_m is the specific capacitance of the device, ΔU is the voltage after IR drop, and Δt is the discharge time.

2. Supplemental Figures

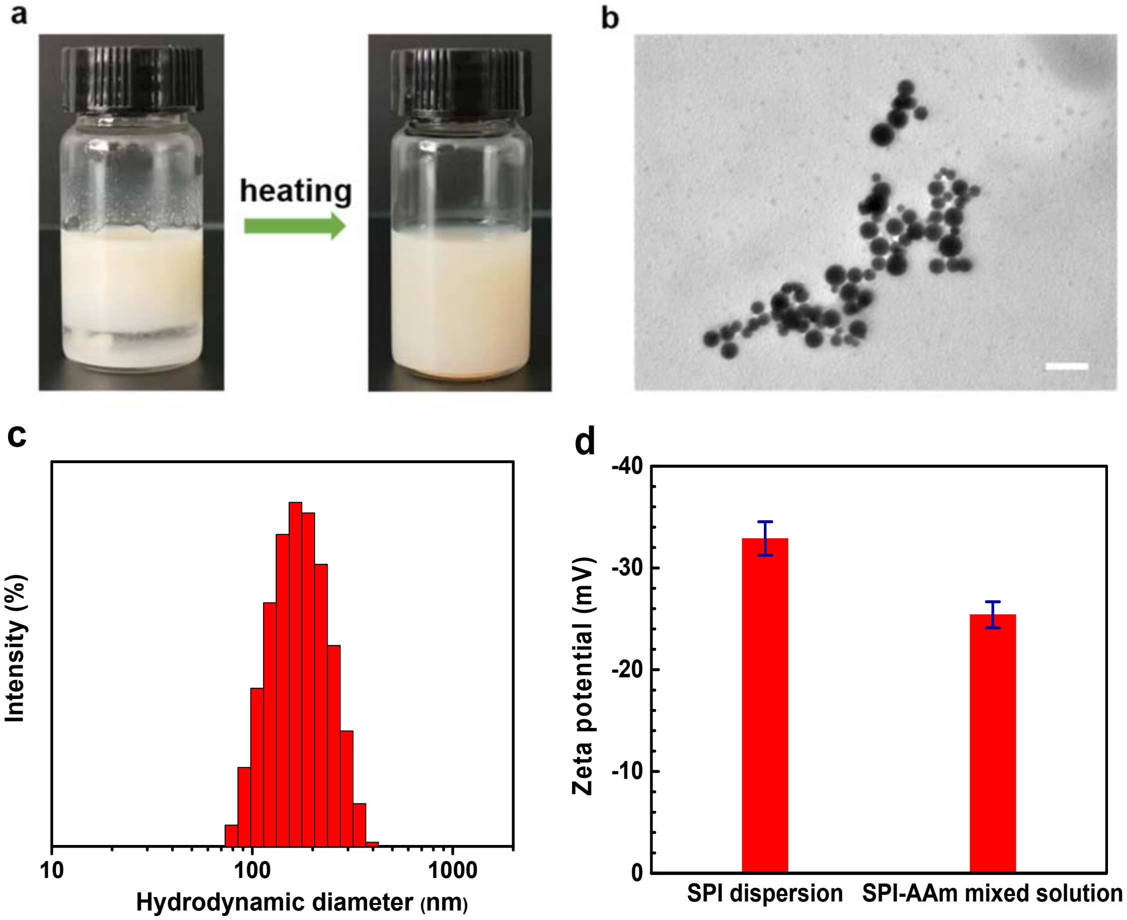

Figure S1. Characterization of SPI nanoparticles. a) Formation of SPI dispersion: SPI powders in water and SPI dispersion after heating treatment. b) TEM image of SPI nanoparticles with an average diameter of ~70 nm. Scale bar: 200 nm. c) Size distribution of SPI dispersion. d) Zeta potentials of SPI dispersion and SPI-AAm mixed solution.

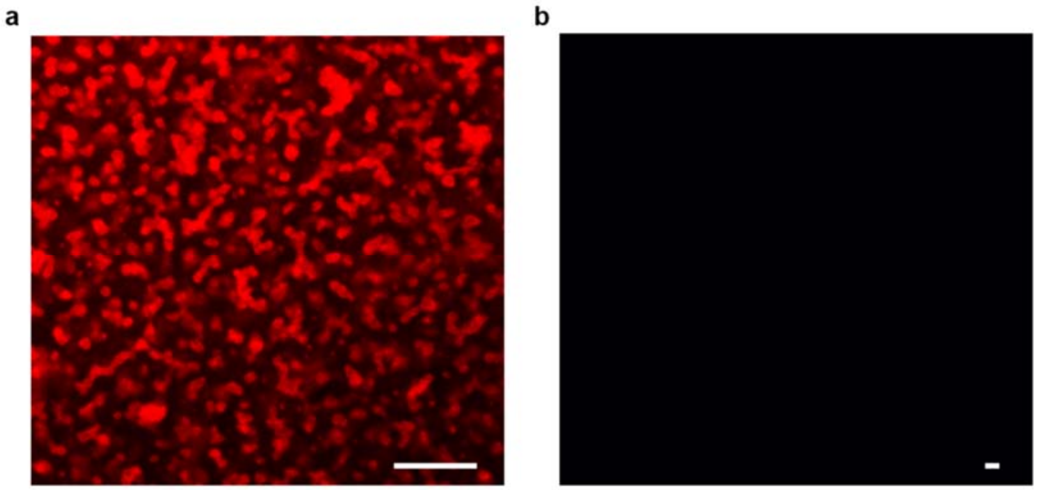

Figure S2. Homogeneity of the hydrogel. Fluorescence images of the hydrogel mixed a) with fluorescent SPI and b) without fluorescent SPI. The scale bars: 10 μm. Fluorescence images show that the fluorescent SPI (in red) are uniformly distributed within the hydrogel.

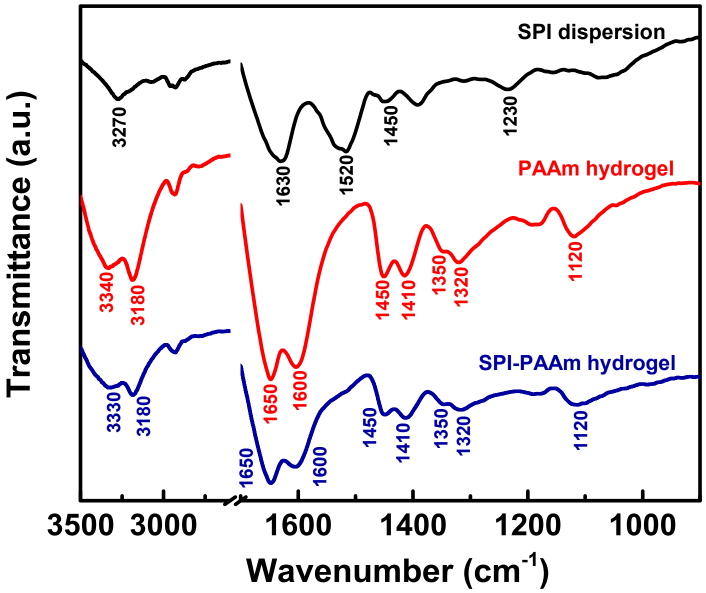

Figure S3. FTIR spectra of SPI dispersion, PAAm hydrogel and SPI-PAAm hydrogel. No new peaks are observed for the hydrogel, indicating that there is no covalent bonding formed between the PAAm chains and SPI nanoparticles.

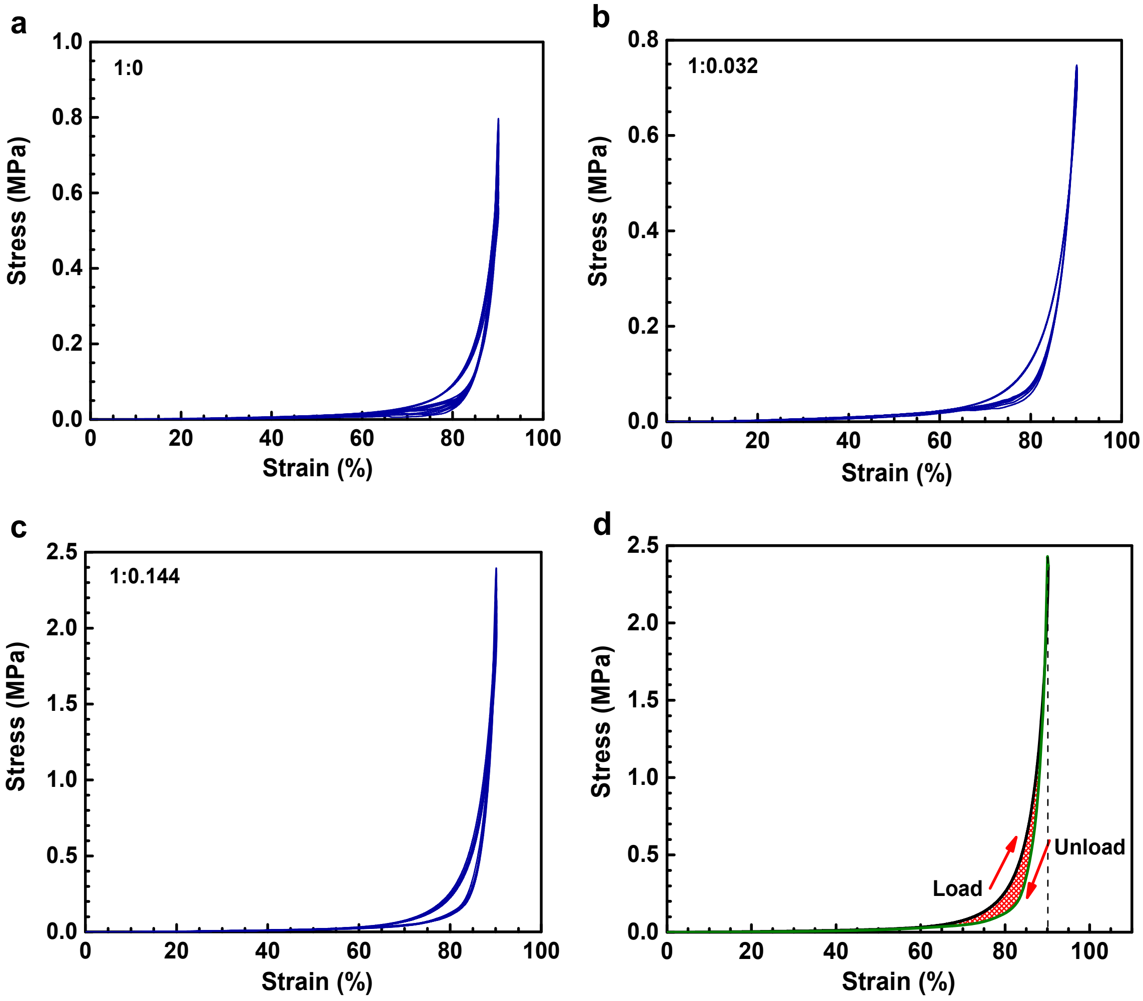

Figure S4. Cyclic compression tests. a-c) Stress-strain curves of hydrogels with different mass ratios of AAm to SPI. d) Stress-strain curve of the hydrogel for one cycle of loading (black line) and unloading (green line). In one cycle, when the stress was unloaded to zero, ε_{min} was the corresponding strain. The plastic deformation = ε_{min}, elastic recovery = 100-ε_{min}; The area beneath the black line (loading curve) gave the total work (U) done during one cycle, the red area (between the loading and unloading curves) was the dissipated energy (E_D), and the area beneath the green line (unloading curve) gave the recovered energy (E_R). The energy loss coefficient = E_D/U, resilience = $E_R/U\times100\%$.

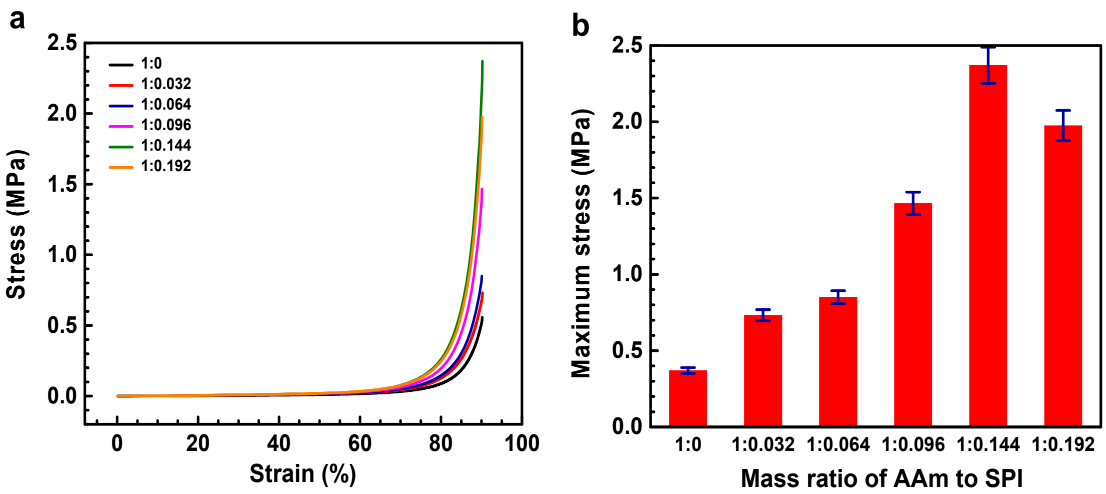

Figure S5. Mechanical properties of hydrogels with different mass ratios of AAm to SPI. a) Compressive stress-strain curves of hydrogels with different mass ratios of AAm to SPI (up to 90% strain). b) The corresponding compressive strength of hydrogels at 90% strain.

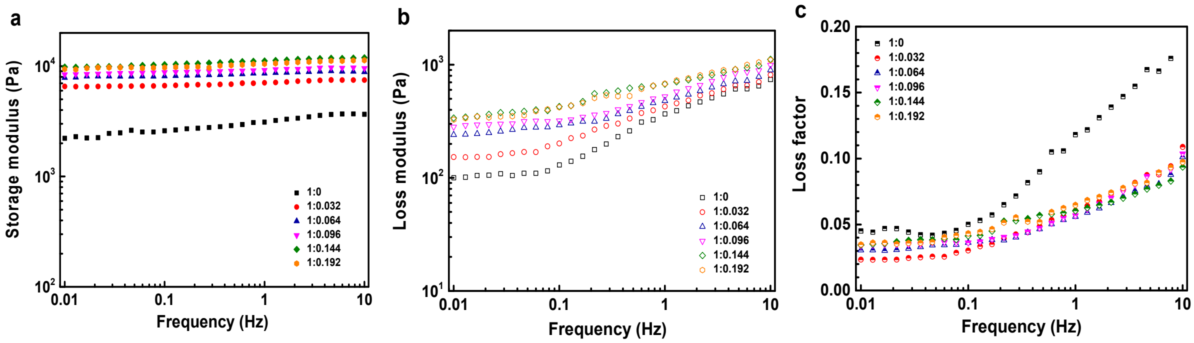

Figure S6. Rheological behaviors of hydrogels with different mass ratios of AAm to SPI. a) Storage modulus. b) Loss modulus. c) Loss factor (the tangential ratio of loss modulus to storage modulus).

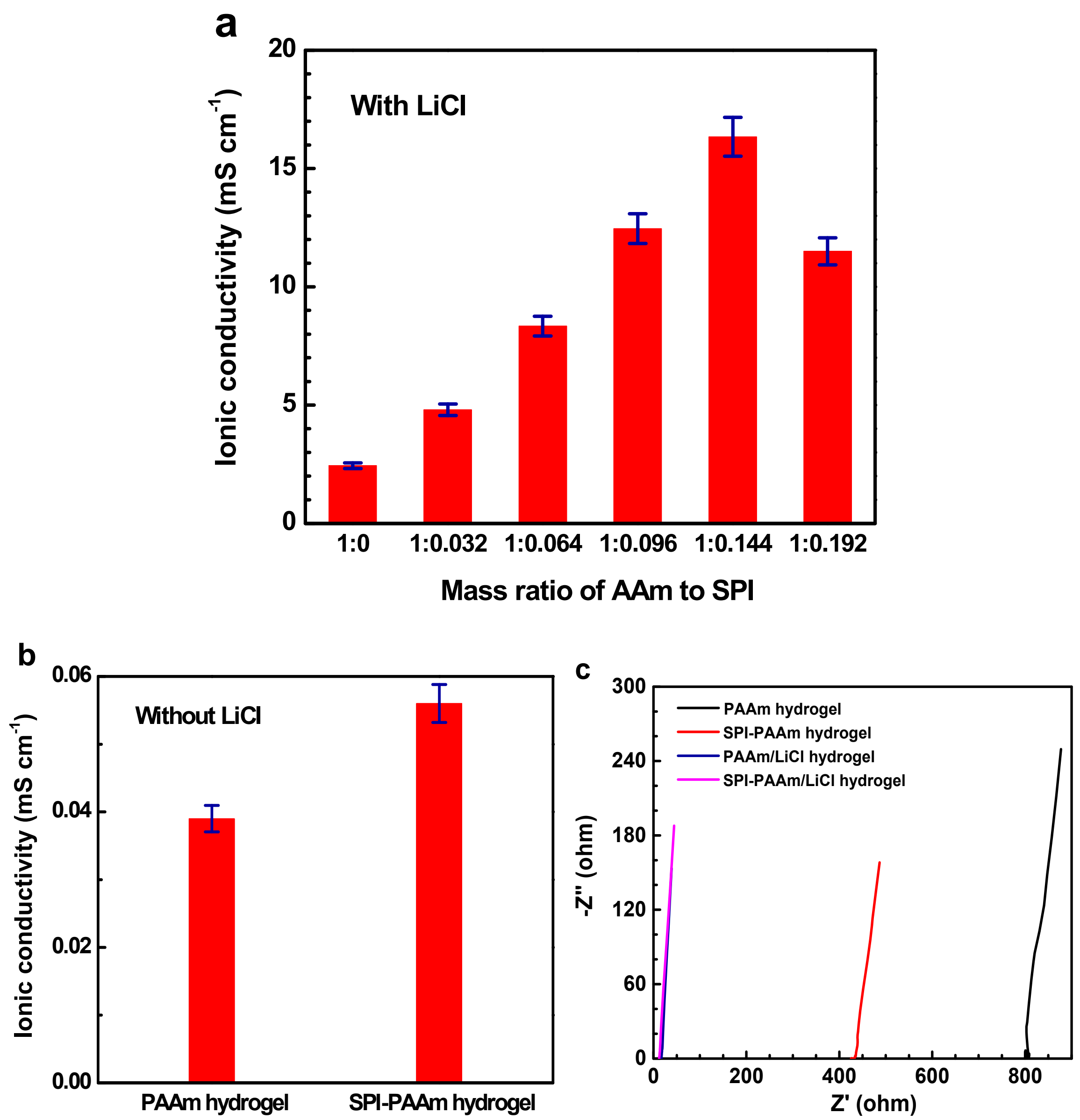

Figure S7. Ionic conductivity and electrochemical properties of hydrogel electrolytes. a) Ionic conductivities of LiCl-containing hydrogels with different mass ratios of AAm to SPI at room temperature. b) Ionic conductivities of PAAm hydrogel and SPI-PAAm hydrogel without adding LiCl salts. c) EIS curves of the devices with different hydrogels as electrolytes. The PAAm hydrogel and SPI-PAAm hydrogel possessed inferior ionic conductivity (Figure S7b), which is two orders of magnitude lower than that of PAAm/LiCl hydrogel and SPI-PAAm/LiCl hydrogel (Figure S7a). The equivalent series resistance (ESR) results (Figure S7c) also reflect the same trend. It indicates that the electrical conductivity of hydrogels is mainly provided by the movable ions in the hydrogel. The insignificant difference in measurements between hydrogels with SPI and without SPI suggests that the SPI colloids have little effect on the ionic and electrochemical properties of hydrogel electrolytes.

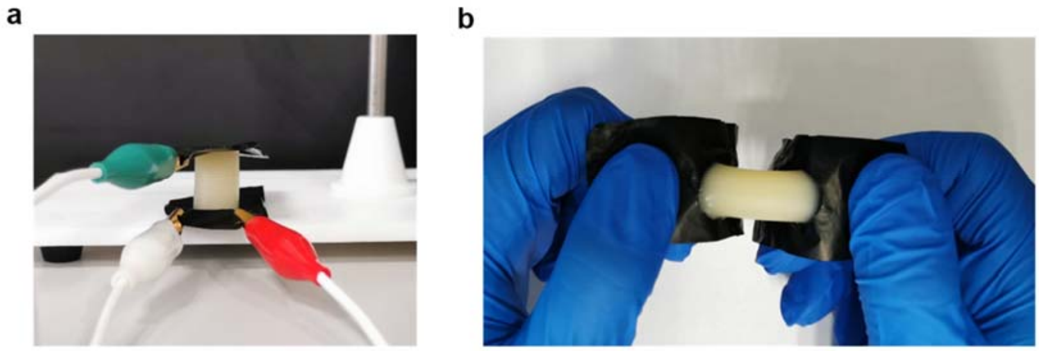

Figure S8. Construction of a quasi-solid-state supercapacitor device. a) Electrochemical testing of the supercapacitors. b) Adhesion between the hydrogel electrolyte and the electrodes.

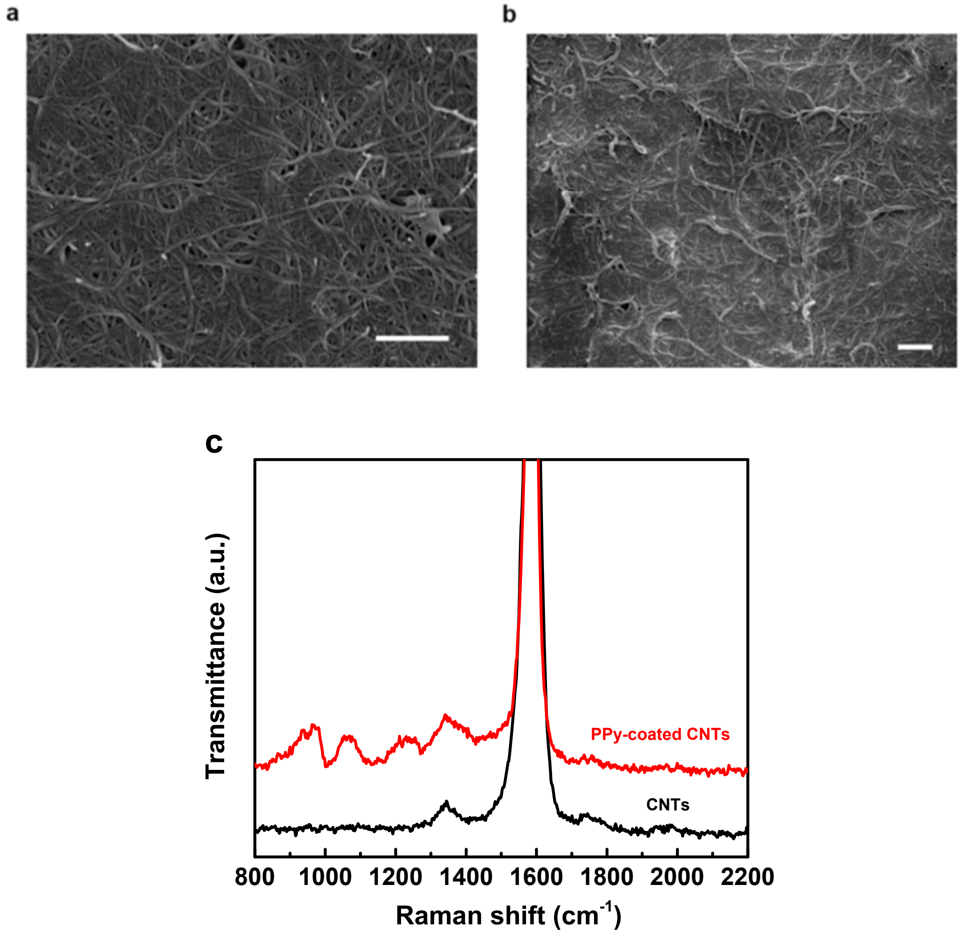

Figure S9. Characterization of PPy-coated CNTs electrode. a-b) SEM images of bare CNTs paper (left) and PPy-coated CNTs paper (right). The scale bars: 500 nm. c) Raman spectra of bare CNTs paper and PPy-coated CNTs paper.

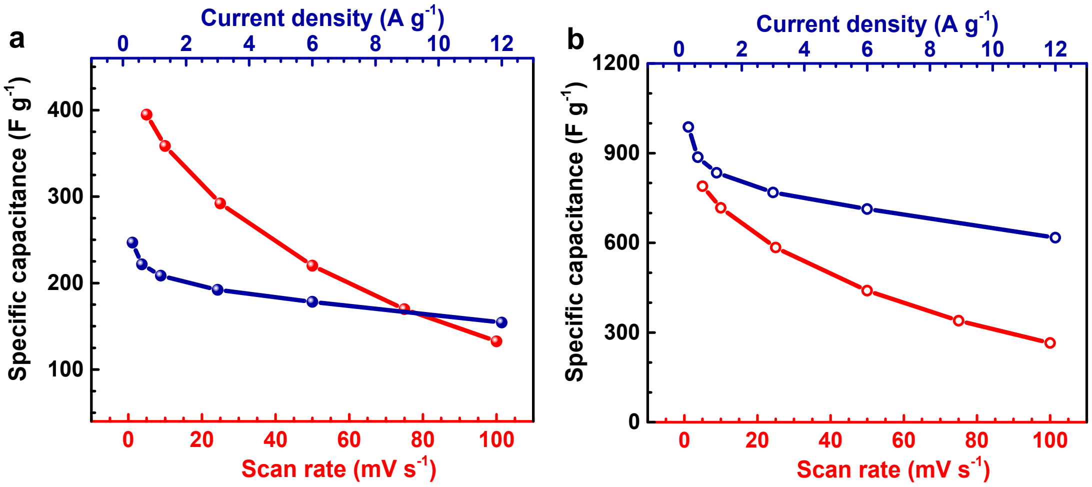

Figure S10. Electrochemical Capacity. a) Capacitances calculated from CVs (red) and GCDs (blue) of the assembled supercapacitor device with the SPI-PAAm hydrogel electrolyte and PPy-coated CNTs electrodes. b) Capacitances calculated from CVs (red) and GCDs (blue) of the single PPy-coated CNTs electrode. CV and GCD curves are shown in the Figure 5a,b.

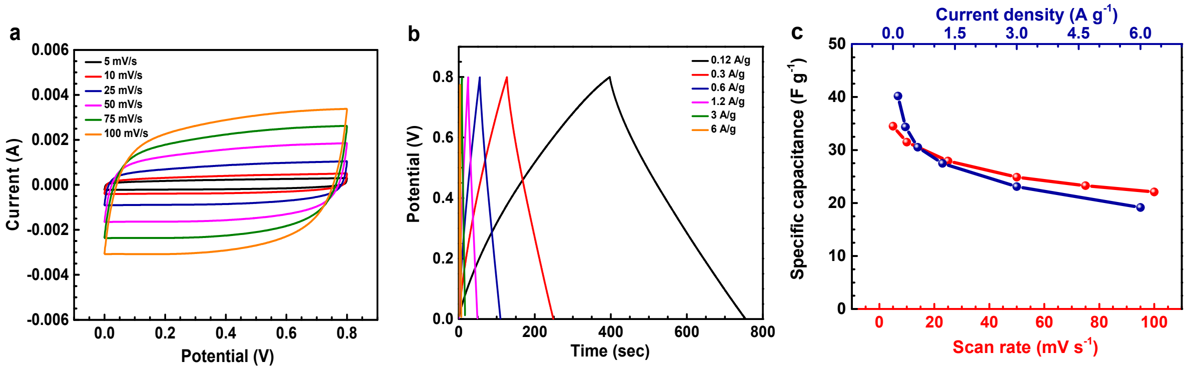

Figure S11. Electrochemical performance of a supercapacitor with bare CNTs electrodes and the SPI-PAAm hydrogel electrolyte. a) CV curves at different scan rates from 5 to 100 mV s^{-1}. b) GCD curves at various charging/discharging current densities from 0.12 to 6 A g^{-1}. c) Capacitances calculated from CVs (red) and GCDs (blue) of the assembled supercapacitor.

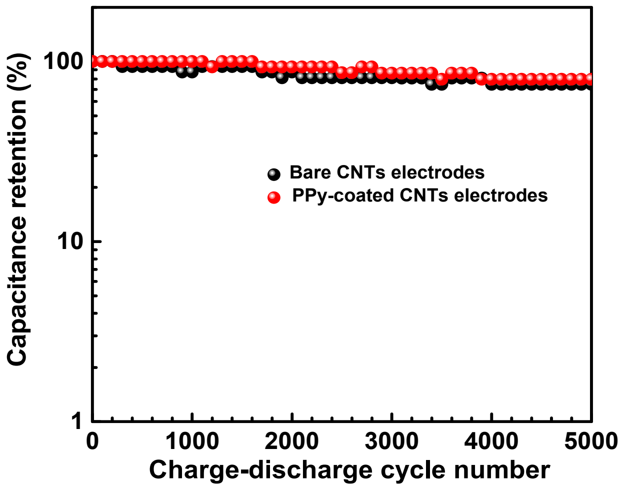

Figure S12. Cycling stability of the supercapacitors. Capacitance retention of the supercapacitor with the SPI-PAAm hydrogel electrolyte and bare CNTs electrodes at a current density of 3 A g^{-1} for 5000 charge/discharge cycles (black sphere), and capacitance retention of the supercapacitor with the SPI-PAAm hydrogel electrolyte and PPy-coated CNTs electrodes at a current density of 10.8 A g^{-1} for 5000 charge/discharge cycles (red sphere).

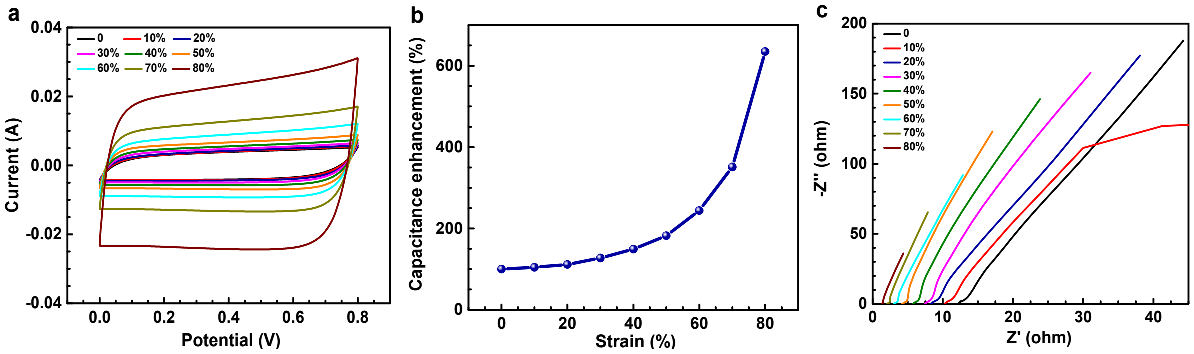

Figure S13. Electrochemical performance of a highly compressible supercapacitor with the SPI-PAAm hydrogel electrolyte and PPy-coated CNTs electrodes. a) CV curves under various strains from 0 to 80% at a scan rate of 50 mV s^{-1}. b) Capacitance enhancement ratios calculated from CVs of (a). c) EIS curves of the device under various compressive strains from 0 to 80%.

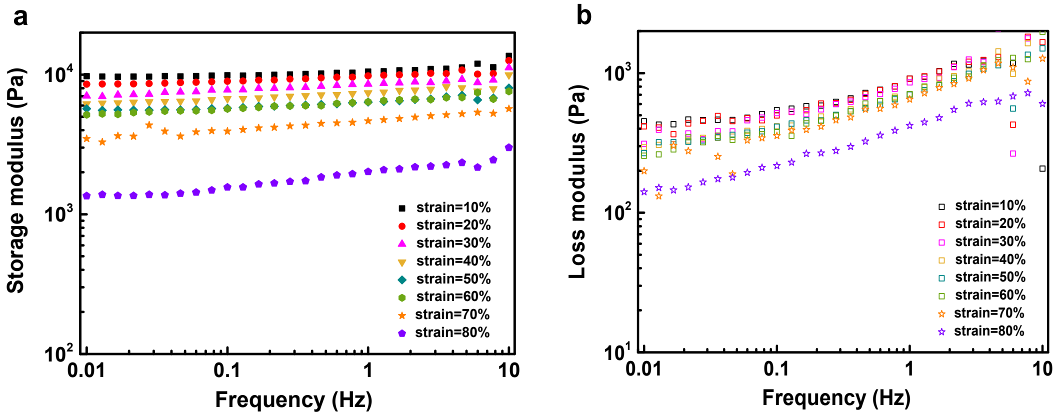

Figure S14. Rheological behaviors of the hydrogel electrolyte at different strains from 0 to 80%. a) Storage modulus. b) Loss modulus.

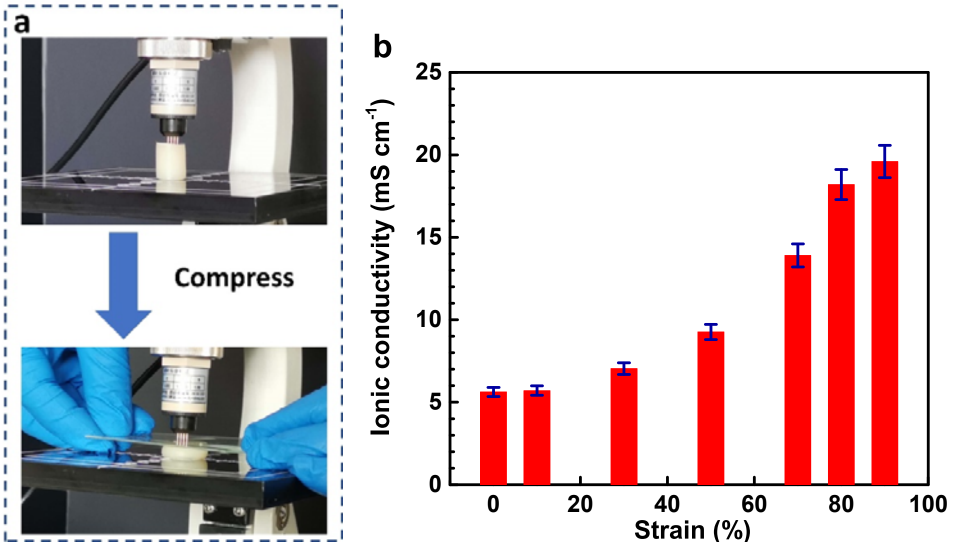

Figure S15. Compression-induced ionic conductivity measurement: (a) Photographs of a hydrogel before and after compression with a four-point probe apparatus placed on the surface of hydrogel; (b) a plot of ionic conductivity vs. strain.

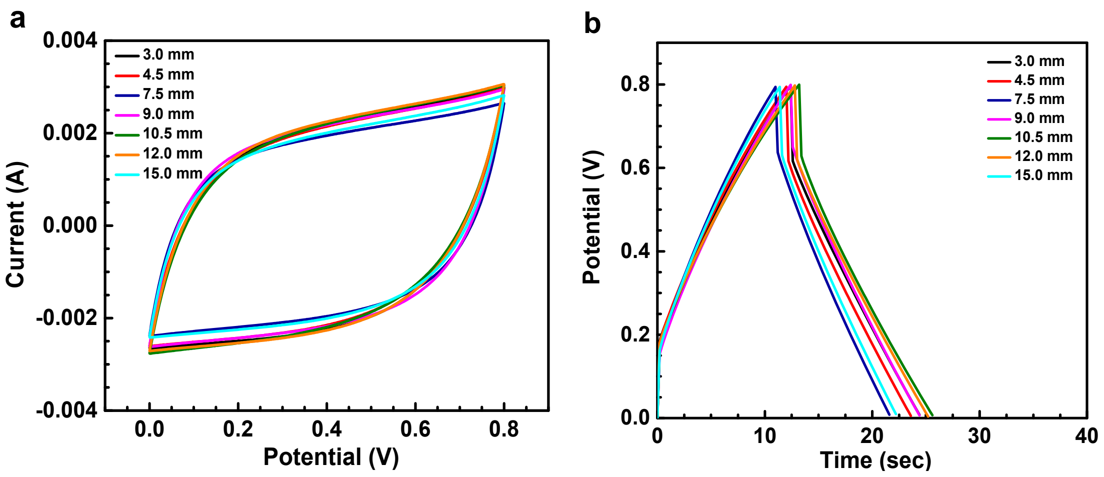

Figure S16. Electrochemical behavior of the assembled supercapacitor device with different hydrogel electrolyte thicknesses. a) CV curves (scan rate: 15 mV/s). b) GCD curves (current density: 6 A/g).

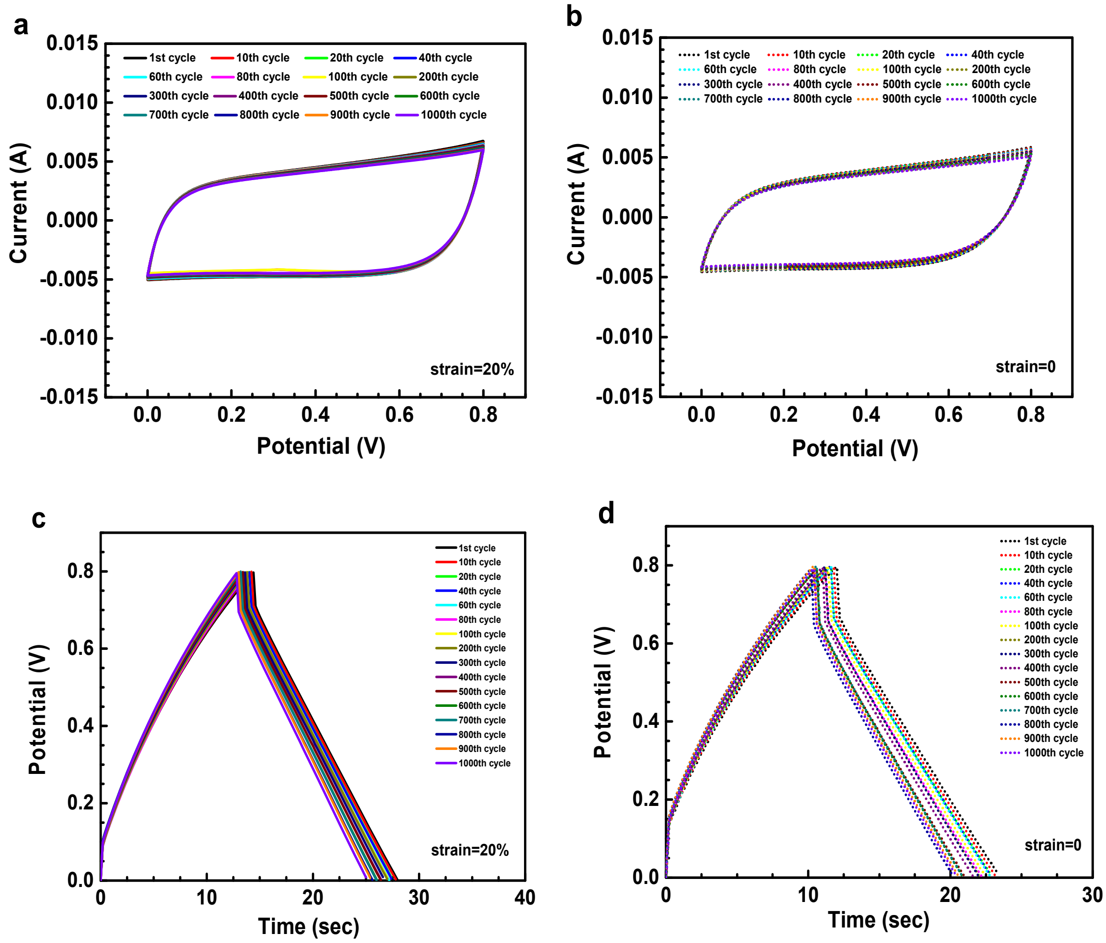

Figure S17. Electrochemical behavior of the assembled supercapacitor device with the SPI-PAAm hydrogel electrolyte and PPy-coated CNTs electrodes at 20% strain for 1000 compression cycles. CVs at different cycle numbers for a) compressed state and b) recovered state. GCDs at different cycle numbers for c) compressed state and d) recovered state.

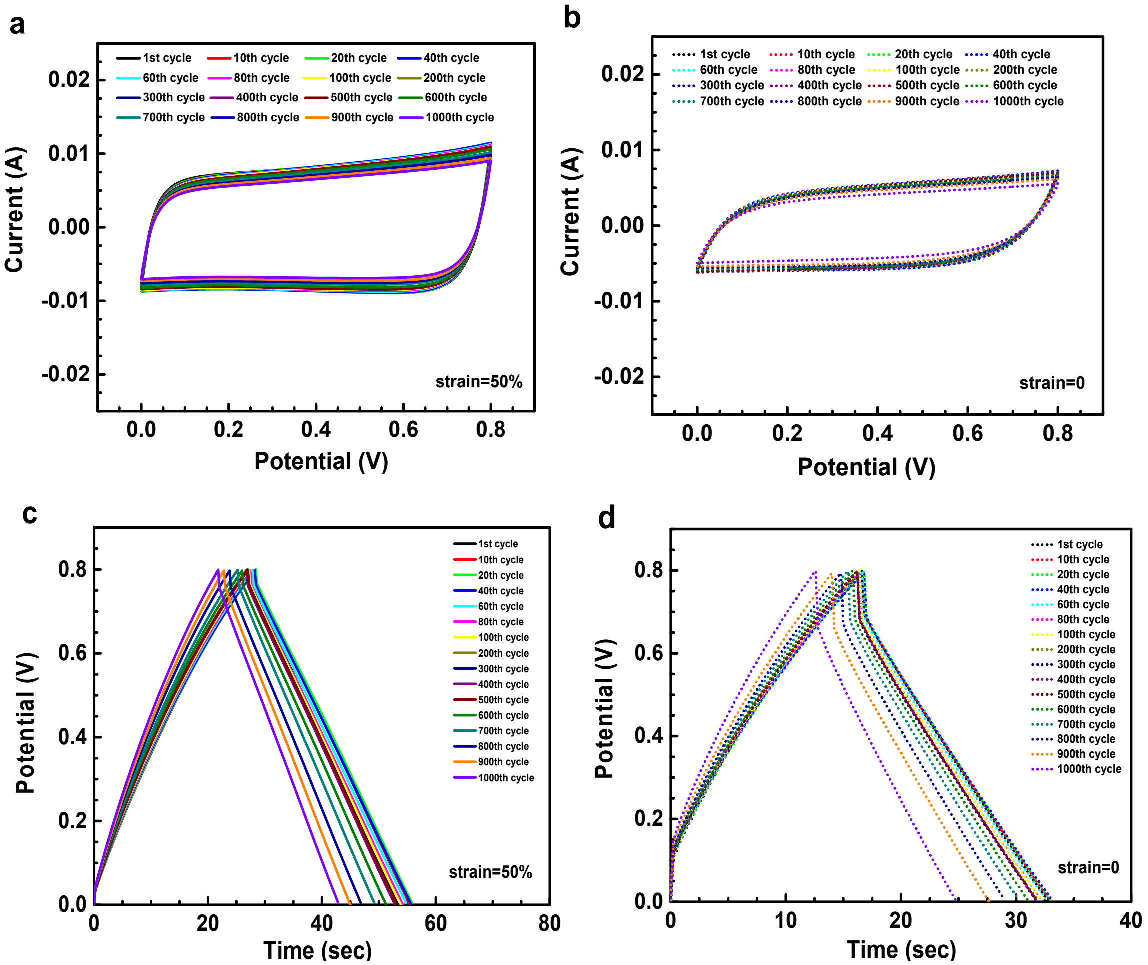

Figure S18. Electrochemical behavior of the assembled supercapacitor device with the SPI-PAAm hydrogel electrolyte and PPy-coated CNTs electrodes at 50% strain for 1000 compression cycles. CVs at different cycle numbers for a) compressed state and b) recovered state. GCDs at different cycle numbers for c) compressed state and d) recovered state.

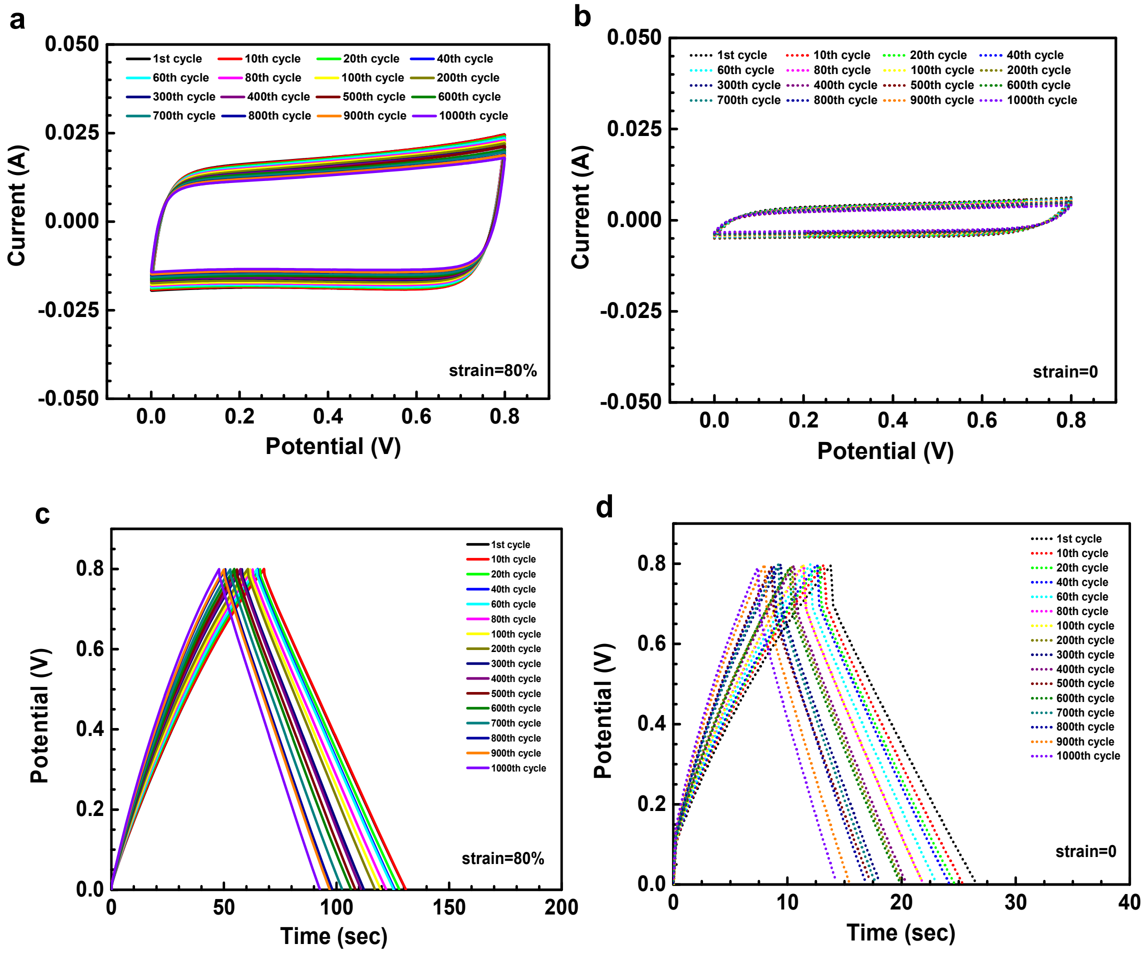

Figure S19. Electrochemical behavior of the assembled supercapacitor device with the SPI-PAAm hydrogel electrolyte and PPy-coated CNTs electrodes at 80% strain for 1000 compression cycles. CVs at different cycle numbers for a) compressed state and b) recovered state. GCDs at different cycle numbers for c) compressed state and d) recovered state.

3. Supplemental Table

Table S1. Comparison of electrochemical performances of compressible supercapacitors.

**Electrode material**	**Electrolyte**	**Specific capacitance**	**Maximum energy density**	**Maximum power density**	**Charge/discharge cycling stability**	**Capacitance retention under compression**	**Ref.**
PPy@CNTs paper	VSNPs-PAM/ H_3PO_4 hydrogel	—	—	—	—	99.4% at 50% compression strain	S1
PPy@CNT paper	VSNPs-PAA hydrogel	—	—	—	—	~300% at 80% compression strain	S2
Activated carbon	EMIMCl/water gel	43.5 F g^{-1} at 0.5 A g^{-1}	—	—	80% after 5000 cycles	300% at 90% compression strain	S3
Nitrogen-doped carbon foam	PVA/LiCl gel	10.3 F g^{-1}at 1 mA cm^{-2}	1.35 Wh kg^{-1}	2900 W kg^{-1}	96% after 4000 cycles	97% at 60% compression strain	S4
Graphene aerogel	KOH/PVA gel	150 F g^{-1} at 0.3 A g^{-1}	10 Wh kg^{-1}	2500 W kg^{-1}	85.1% after 10000 cycles	98% at 75% compression strain<br>85% after 100 compression cycles at 50% strain	S5
PANI-SWCNTs-sponge	PVA/H_2SO_4 gel	216 F g^{-1}	21 Wh kg^{-1}	1500 W kg^{-1}	92% after 1000 cycles	97% at 60% compression strain<br>stable after 100 compression cycles at 60% strain	S6
PPy-CNTs	PAA hydrogel	0.22 mF cm^{-2} at 5 mV s^{-1}	—	—	—	stable at 60% compression strain	S7
CNC-MWCNT-PPy aerogel	Na_2SO_4 aqueous	43.7 F g^{-1} at 2 mV s^{-1}	5.5 Wh kg^{-1}	2000 W kg^{-1}	86.9% after 5000 cycles	104.7% at 80% compression strain	S8
PEI/CNT	Na_2SO_4 aqueous	25 F g^{-1}	1 Wh kg^{-1}	60 W kg^{-1}	75% after 400 cycles	~100% at 75% compression strain	S9
PPy-graphene foam	$NaClO_4$ aqueous	350 F g^{-1} at 1.5 A g^{-1}	—	—	~100% after 1000 cycles	~100% at 50% compression strain<br>no change after 1000 compression cycles at 50% strain	S10
CNT sponge	KCl aqueous	28.5 F g^{-1} at 1 mV s^{-1}	—	—	94% after 15000 cycles	65% at 90% compression strain<br>96% after 1000 compression cycles at 50% strain	S11

CNT@PPy@MnO_2 sponge	KCl aqueous	305.9 F g^{-1} at 2 mV s^{-1}	8.5 Wh kg^{-1}	16.5 kW kg^{-1}	90.2% after 1000 cycles	90% at 90% compression strain	S12
CNT@PPy sponge	KCl aqueous	376 F g^{-1} at 0.5 A g^{-1}	—	—	80% after 1000 cycles	90% at 50% compression strain<br>98% after 1000 compression cycles at 50% strain	S13
CNT-graphene aerogel	Na_2SO_4 aqueous	70 F g^{-1}	—	—	97% after 400 cycles	~100% at 90% compression strain	S14
CNT@Mesoporous carbon@Pt sponge	KOH aqueous	207 F g^{-1}	4.6 Wh kg^{-1}	3000 W kg^{-1}	125% after 5000 cycles	96% at 50% compression strain	S15
rGO aerogel	H_2SO_4 aqueous	90 F g^{-1} at 10 mV s^{-1}	—	—	84.5% after 2000 cycles	144% at 90% compression strain<br>~95% after 1000 compression cycles at 50% strain	S16
PPy-coated CNTs paper	SPI-PAAm/LiCl hydrogel	246.8 F g^{-1} at 0.3 A g^{-1}	21.4 W h kg^{-1}	2580 W kg^{-1}	~80% after 5000 cycles	628% at 80% compression strain<br>90% for 1000 compression cycles at 20% strain; 80% for 1000 compression cycles at 50% strain; 57% for 1000 compression cycles at 80% strain	This work

References

[S1] Y. Huang, M. Zhong, F. Shi, X. Liu, Z. Tang, Y. Wang, Y. Huang, H. Hou, X. Xie, C. Zhi, *Angew. Chem. Int. Ed.* **2017**, *56*, 9141.

[S2] Y. Huang, M. Zhong, Y. Huang, M. Zhu, Z. Pei, Z. Wang, Q. Xue, X. Xie, C. Zhi, *Nat. Commun.* **2015**, *6*, 10310.

[S3] X. Liu, D. Wu, H. Wang, Q. Wang, *Adv. Mater.* **2014**, *26*, 4370.

[S4] K. Xiao, L. X. Ding, G. Liu, H. Chen, S. Wang, H. Wang, *Adv. Mater.* **2016**, *28*, 5997.

[S5] D. Jiang, C. Li, W. Yang, J. Zhang, J. Liu, *J. Mater. Chem. A* **2017**, *5*, 18684.

[S6] Z. Niu, W. Zhou, X. Chen, J. Chen, S. Xie, *Adv. Mater.* **2015**, *27*, 6002.

[S7] M. Hu, J. Wang, J. Liu, J. Zhang, X. Ma, Y. Huang, *Chem. Commun.* **2018**, *54*, 6200.

[S8] K. Shi, X. Yang, E. D. Cranston, I. Zhitomirsky, *Adv. Funct. Mater.* **2016**, *26*, 6437.

[S9] G. Nyström, A. Marais, E. Karabulut, L. Wågberg, Y. Cui, M. M. Hamedi, *Nat. Commun.* **2015**, *6*, 7259.

[S10]Y. Zhao, J. Liu, Y. Hu, H. Cheng, C. Hu, C. Jiang, L. Jiang, A. Cao, L. Qu, *Adv. Mater.* **2013**, *25*, 591.

[S11]P. Li, C. Kong, Y. Shang, E. Shi, Y. Yu, W. Qian, F. Wei, J. Wei, K. Wang, H. Zhu, *Nanoscale* **2013**, *5*, 8472.

[S12]P. Li, Y. Yang, E. Shi, Q. Shen, Y. Shang, S. Wu, J. Wei, K. Wang, H. Zhu, Q. Yuan, *ACS Appl. Mater. Interfaces* **2014**, *6*, 5228.

[S13]J. Y. Sun, C. Keplinger, G. M. Whitesides, Z. Suo, *Adv. Mater.* **2014**, *26*, 7608.

[S14]E. Wilson, M. F. Islam, *ACS Appl. Mater. Interfaces* **2015**, *7*, 5612.

[S15]Y. Yang, P. Li, S. Wu, X. Li, E. Shi, Q. Shen, D. Wu, W. Xu, A. Cao, Q. Yuan, *Chem. Eur. J.* **2015**, *21*, 6157.

[S16]J. Y. Hong, B. M. Bak, J. J. Wie, J. Kong, H. S. Park, *Adv. Funct. Mater.* **2015**, *25*, 1053.
